# Evaluation of Minimum Inhibitory Concentration of Heavy Metals Contained in Packaging Material Digest on Prominent Gut Microbiota

**DOI:** 10.1155/2023/3840795

**Published:** 2023-11-21

**Authors:** Senna Mukhi, Biranthabail Dhanashree, Rukmini Mysore Srikantiah, Poornima Manjrekar, Sindhu Harish

**Affiliations:** ^1^Department of Biochemistry, Kasturba Medical College, Mangalore, Manipal Academy of Higher Education, Manipal, India; ^2^Department of Microbiology, Kasturba Medical College, Mangalore, Manipal Academy of Higher Education, Manipal, India

## Abstract

Several scientific investigations have revealed that the leaching of metals from packaging material into the packed food is an unavoidable process. Hence, this study is aimed at investigating the effect of leached heavy metals from food packing materials on normal human gut flora. We analysed the effect of vanadium, arsenic, cadmium, and mercury present in digested packaging materials (DPM) on standard strains of *Escherichia coli* ATCC 25923, *Pseudomonas aeruginosa* ATCC 27853, *Klebsiella pneumoniae* ATCC 70063, and *Enterococcus faecalis* ATCC 29212. The minimum inhibitory concentration (MIC) of laboratory-grade heavy metal salts and heavy metals present in DPM was determined by the agar dilution method. For all four bacteria, the MIC of cadmium and arsenic in the DPM was 7 *μ*g/ml and 1.6 *μ*g/ml, respectively. The MIC of mercury in DPM was 1.6 *μ*g/ml for *E. coli*, *K. pneumoniae*, and *E. faecalis* and 1.4 *μ*g/ml for *P. aeruginosa*. MIC of vanadium for *E. coli*, *P. aeruginosa*, and *E. faecalis* was 2.2 *μ*g/ml, and for *K. pneumoniae* was 2.0 *μ*g/ml. The difference in MICs of heavy metals in DPMs and heavy metal salts was not statistically significant. MICs were within CODEX-specified permissible levels. Though heavy metals in packaging material have not shown a deleterious effect on representative human gut flora, there is scope to study their effect on the gut microbiome. Thus, understanding the risk of heavy metal ingestion through unknown sources and avoiding any possible ingestion is crucial to preventing chronic diseases.

## 1. Introduction

Packaging material is any substance or article that will be in continuous contact with the food and drug. These include containers such as bottles, cans, boxes, cases, and cartons and casing materials such as film, foil, metal, paper, cloth, or wax paper [1]. The major reason for packaging is to offer protection from foreign materials during transportation and to maintain the shelf life of the food product. Packaging can be deceptive and expensive, adding to the inadvertent use of natural resources and paving the way for the accumulation of nonbiodegradable waste and pollution [2]. Packaging material has become one of the most important elements in the food industry, and various types of additives such as stabilizers, antioxidants, lubricants, antiblockers, and antistatic agents are used to improve storage. According to research, chemicals, phthalates, bisphenols, monomers, heavy metals, and other toxins can enter food through packaging [3]. In the context of food packaging, the term leaching is defined as the migration of particles from the surface of the packaging material to the food/drug [4].

Heavy metals may be present in the core packaging material due to its manufacturing process or in the food dispensed or unintentionally added at a specific step of the production of food or food packages [5–8]. Although there are quality evaluations and testing of packaging material before the food is released into the market, there is a paucity of studies undertaken to analyse the leaching of toxic metals into a particular type of food and medication and their impact on human health [9]. The Codex Alimentarius Commission (CAC), established by the Food and Agriculture Organization (FAO) of the United Nations in 1961, has set goals along with the World Health Organization (WHO) to safeguard the well-being of consumers and verify fair-minded practices in the food trade [10]. In addition, standards for specific foods, food labelling, hygiene, additives, insecticide residues, and procedures are set for evaluating the safety of food [11]. According to the regulations, the maximum permissible limit of vanadium (V) in food is 1.8 *μ*g/ml, cadmium (Cd) is 7 *μ*g/ml, mercury (Hg) is 1.4 *μ*g/ml, and arsenic (As) is 1.6 *μ*g/ml [12].

The minimum inhibitory concentration (MIC) is the lowest concentration of an agent that inhibits the observable growth of a microorganism after overnight incubation. Mainly, diagnostic laboratories use MICs to detect antimicrobial resistance breakpoints and as a research tool to detect the *in vitro* activity of new antimicrobials [13, 14]. A study by Sood and Sharma demonstrated fourteen different heavy metals in ten commonly used food packaging materials [15]. The leaching of chemicals such as colourants, adhesives, metals, and polymers into the packed food has been shown to have a deleterious effect on the gut flora [16]. Healthy gut flora is necessary for the overall health of humans [17]. Hence, the present study is aimed at knowing the MIC of heavy metals present in digests of commonly used packaging materials for *Escherichia coli* ATCC 25923, *Pseudomonas aeruginosa* ATCC 27853, *Klebsiella pneumoniae* ATCC 70063, and *Enterococcus faecalis* ATCC 29212. These strains were chosen as representative members of the human gut flora to know whether the heavy metals present in DPM are harmful to the gut flora and are at CAC permissible levels.

## 2. Materials and Methods

### 2.1. Study Setting

The study was done at the Department of Microbiology, Kasturba Medical College, Mangalore, with the approval of the institutional ethics committee (ethical clearance reference number: IEC KMC MLR 04-2021/131).

### 2.2. Heavy Metal Salts Used and Stock Solution Preparation

Laboratory-grade heavy metal salts of vanadium (vanadium pentoxide), arsenic (arsenic trioxide), cadmium (cadmium acetate), and mercury (mercuric chloride) with purity ranges of 98.5 to 99.9% were procured from Intelligent Materials, Pvt. Ltd., Nanoshel Group of Companies, Punjab, India. Each of these salts of heavy metals was dissolved in sterile distilled water for the preparation of a 100 *μ*g/ml stock solution. This was sterilized by autoclaving and incorporated into Luria Bertani (LB) agar procured from Hi Media Laboratories, Thane -400604, Maharashtra, India (Table 1).

### 2.3. Preparation of Digest of Packaging Material (DPM)

Initially, 13 different types of commonly used food and drug packaging materials such as aluminium cans, leak-proof bags, cardboard, tetra packs, cellophane, tissues, sachets, aluminium bags and boxes, plastic bags and containers, medicinal blister packets, and medicinal closures (one each weighing 10 grams) were subjected to microwave-assisted digestion. Qualitative and quantitative analysis by Inductively Coupled Plasma Optical Emission Spectroscopy (ICP-OES) (Agilent Technologies, Santa Clara, California, USA) showed that digests of cardboard, sachet, and medicinal closure contained the highest quantity of heavy metals. Hence, we chose to analyse five samples each of cardboard, sachet, and medicinal closure. Digestion of 10 grams each of cardboard, sachet, and medicinal closure was done by microwave-assisted digestion as per the USEPA 3051 guidelines [18]. Briefly, the packaging materials were cut into small pieces and individually dissolved in concentrated nitric acid, followed by concentrated hydrochloric acid, in a laboratory microwave unit. The sample with the acids was placed in a quartz microwave vessel and sealed. Sealed vessels containing medicinal closures, cardboard, and sachets were heated in the microwave for 120, 90, and 45 minutes, respectively. The vessels were cooled, and the contents were filtered, centrifuged, and diluted using distilled water. The pH of the digest was adjusted to 7.4. ICP-OES was used to quantify the amount of heavy metal present in each of the DPM. Analytical grade reagents, chemicals (Sigma-Aldrich, Taufkirchen, Germany), and double-distilled water were used for the preparation of solutions and dilutions.

### 2.4. Sterility of the DPM

The digests of cardboard, sachets, and medicinal closures containing heavy metals were sterilized by autoclaving at 121°C for fifteen minutes. The concentration of respective heavy metals in the DPMs was determined by ICP-OES and dissolved in sterile distilled water to bring the concentration to 100 *μ*g/ml. This was used as a stock solution for preparing the Luria Bertani (LB) agar. Since the digest of cardboard had 2.7 *μ*g/ml of vanadium, it was dried in a hot air furnace at 80°C to evaporate the solvent and increase the concentration. The concentrated DPM of cardboard containing vanadium was dissolved in distilled water to obtain the stock solution with a concentration of 100 *μ*g/ml. These stock solutions of heavy metal digests of cardboard, sachets, and medicinal closures were used to study the minimum inhibitory concentration (MIC) on standard strains of bacteria that represent gut flora.

### 2.5. Bacterial Cultures and Media

Standard strains of *E. coli* ATCC 25923, *P. aeruginosa* ATCC 27853, *K. pneumoniae* ATCC 70063, and *E. faecalis* ATCC 29212 available in the microbiology department stock culture collection were used in the study. Luria Bertani (LB) agar was incorporated with the different concentrations of sterile digest of packaging material (DPM) and heavy metal salt solution, as shown in Table 1. LB agar was autoclaved, poured into 10 cm Petri plates, and allowed to set. These LB agar plates were used for determining the MIC of heavy metals.

### 2.6. Controls

Sterile LB agar incorporated with varying concentrations of heavy metal salt and DPMs was used as a sterility control (blank). LB agar without heavy metal, streaked with the standard strain of bacteria, was used as a growth control (positive control).

### 2.7. Detection of MIC

LB agar containing varying concentrations of the DPMs with V, Hg, As, and Cd and heavy metal salt solutions was used to study the MIC. Overnight cultures of standard strains of bacteria whose turbidity was adjusted to 0.5 McFarland standards (1 × 105 CFU/mL) were spot inoculated (2 *μ*l) on the LB agar plates containing various concentrations of DPMs containing heavy metals/heavy metal salts (Table 1). LB agar plates with various concentrations of heavy metal salt/DPMs, without the bacterial inoculum, were used as sterility controls (blank). LB agar plates without heavy metal salt and DPMs were inoculated with different ATCC bacterial strains used as growth control (positive control). The entire inoculated and control LB agar plates were incubated at 37°C for 48 hr. Observed for bacterial growth at the end of 24 and 48 hr intervals, and the results were noted down [19–21]. Pictures of Luria bertani agar, different concentrations of heavy metal salts, and DPM are shown in figures S1 to S4 in the supplementary file.

### 2.8. Statistical Analysis

Experiments were performed in triplicate. The mean of the triplicates was taken as a result. Statistical analysis was done using Microsoft Excel (Version 2308). The mean MIC value of the heavy metal salts was used as the standard. The mean MIC value of DPM was compared with the mean MIC of standard heavy metals. The standard deviation was also calculated. A difference in MIC with a *p* value < 0.05 is considered significant.

## 3. Results and Discussion

### 3.1. Quantification of Heavy Metals in Packaging Materials

Qualitative and quantitative analysis of DPMs by ICP-OES showed that cardboard, sachets, and medicinal closures had the highest quantity of heavy metals among the 13 packaging materials analysed. The remnants of food/drugs if present in the packaging material got degraded during heating (60°C-80°C) for a prolonged period of time, and acid treatment was used for their digestion process. Digest of cardboard had 2.7 *μ*g/ml of vanadium, sachets had 155 *μ*g/ml of cadmium and 213 *μ*g/ml of mercury, and medicinal closure had 101.41 *μ*g/ml of arsenic. These concentrations of heavy metals in the DPMs were higher than the permissible levels specified by CAC which is cause for concern. CAC acceptable values for vanadium are 1.8 *μ*g/ml, cadmium 7 *μ*g/ml, mercury 1.4 *μ*g/ml, and arsenic 1.6 *μ*g/ml [12]. Earlier studies have shown the presence of heavy metals in various packaging materials that are the mainstay of the modern food industry [15, 16]. Even the present study has detected vanadium, arsenic, cadmium, and mercury in the digests of cardboard, sachets, and medicinal closures. The study by Yousi et al. reports that many food packaging materials contain vanadium, mercury, arsenic, and cadmium that leach out at low levels and are known to cause diarrhoea, nausea, and vomiting [22]. However, they have used the microbiome of faecal transplant donors, and our study focuses on four standard strains of bacterial cultures.

### 3.2. MIC of Heavy Metal Salts and Digests of Cardboard, Sachets, and Medicinal Closures

As a normal gut flora, *E. coli*, *K. pneumoniae*, *E. faecalis*, and *P. aeruginosa* are beneficial for human health [23–25]. Hence, we selected them as representative gut flora to study the MIC of heavy metals present in DPM. Using LB agar, we studied the MIC of heavy metal salts, namely, vanadium pentoxide, arsenic trioxide, cadmium acetate, and mercuric chloride, whose purity ranged from 98.5 to 99.9%, and compared these with the MICs obtained for V, Cd, Hg, and As in the digests of cardboard, sachets, and medicinal closures. LB agar plates with various concentrations of heavy metal salts/DPM without bacterial inoculum were sterile. LB agar plates without heavy metals inoculated with various ATCC bacterial strains showed good growth, which indicated that the batch of LB agar prepared supported the bacterial growth.  Table 2 depicts the purity and MIC of heavy metal salts for *E. coli* ATCC 25923, *P. aeruginosa* ATCC 27853, *K. pneumoniae* ATCC 70063, and *E. faecalis* ATCC 29212. This MIC of heavy metal salt with a purity of 98% to 99% was taken as a standard to compare the MIC of heavy metals in DPMs [26–29].  Table 3 shows the yield of heavy metals from digests of cardboard, sachets, and medicinal closures, the CAC permissible level of heavy metals in DPMs, and their MIC for standard strains of bacteria tested.

The CAC permissible limit for vanadium is 1.8 *μ*g/ml. However, the MIC of vanadium from the extract of cardboard for all four standard bacteria tested is higher than the specified limit, which indicates no risk to the bacteria tested. The CAC limit for cadmium is 7 *μ*g/ml. The MIC of Cd was 7 *μ*g/ml for *E. coli*, *E. faecalis*, and *K. pneumoniae* and 7.2 *μ*g/ml for *P. aeruginosa*. Since the MIC of cadmium in the DPMs is within the CAC levels, it may not be harmful to healthy gut microbiota even if it leaches. The CAC limit set for mercury is 1.4 *μ*g/ml, and the MIC of mercury for *E. coli* was found to be 1.6 *μ*g/ml, and for *E. faecalis*, *K. pneumoniae*, and *P. aeruginosa*, it was 1.4 *μ*g/ml. Hence, the CAC permissible limit for mercury is also considered safe. MIC of arsenic for *E. faecalis* was 1.4 *μ*g/ml, and for all the other three bacteria, it was found to be 1.6 *μ*g/ml, which is equal to the CAC limit. Thus, the MIC of four heavy metals present in the DPMs was within the CAC permissible levels (Table 3). Earlier workers have observed the suppression of growth of these gut microorganisms by concentrations of heavy metals lower than the CAC permissible level, indicating a harmful effect of heavy metals on the normal flora [24, 29, 30]. Thus, our findings are contradictory to earlier findings as their study settings have used animal models, cell cultures, or human microbiota from transplant donors.

Leached heavy metals may harm the gut microbiota, which helps to regulate the entire function of the human gut. The study by Balali-Mood et al. shows that heavy metal ingestion is harmful to humans and can lead to diseases such as dysfunctions of the gastrointestinal tract, kidneys, and immune system, nervous system disorders, skin lesions, vascular injury, birth flaws, and cancer at higher ingestion [30].

We compared the mean MIC of the four heavy metal salts (98-99% pure) and DPMs (purity: 4.08%-24.60%) to know whether there is any statistically significant difference using GraphPad software V.10.0.2 (La Jolla, California, United States). Only a marginal difference was observed in the mean MICs, as shown in Tables 2 and 3 which was not statistically significant (unpaired *t* test; *p* = 0.9532 and paired *t* test *p* = 0.1881; *p* value < 0.05 is significant). However, concentrations of V, Hg, As, and Cd content in the packaging materials studied were higher than the CAC permissible levels and did not have any hazardous effect on the gut flora tested. However, if leached heavy metal is ingested with food, it may accumulate in the tissues of animals and humans. This bioaccumulation may have negative impacts from heavy metal toxicity on the biota of riverine habitats [31]. Thus, bioaccumulation can lead to harmful diseases in humans such as dementia, neurodegenerative disorders, and neurodevelopmental impacts such as Alzheimer's and Parkinson's disease [32]. Hence, there is a need to avoid heavy metals in packaging materials or use biodegradable, environmentally friendly packaging materials.

The limitation of this study is that we did not study the quantity of metals leached into stored food. Therefore, further studies are required to focus on the time of contact of products with food packaging material and the quantity of metals leached within the limits of CAC standards. There is also a need for the study to evaluate the MIC of a wide range of heavy metals present in food packaging material on the entire human microbiome and human gut cell lines.

## 4. Conclusions

The MIC levels of vanadium, cadmium, arsenic, and mercury, when tested on representative gut bacteria, were found to be within the permitted limits set by regulatory authorities. However, the content of heavy metals in the DPM was higher than the CAC-permissible levels. As long as they are not leached into the food, these heavy metals may not pose an immediate threat to gut microbiota and human health. Nevertheless, further research is warranted to comprehensively understand the long-term effects of these heavy metals on the gut microbiome of healthy individuals. While the initial findings are reassuring, it is advisable to exercise caution when it comes to packaging materials containing heavy metals, given their well-documented hazards to human health. As a safer alternative, opting for reusable or biodegradable packaging materials, such as those made from leaves, for packing food items is a responsible choice that aligns with both environmental and health considerations.

## Figures and Tables

**Table 1:** Luria Bertani agar with different concentrations of digests of packaging material/heavy metal salt solution for the detection of minimum inhibitory concentration.

Name of heavy metal in the digests of packaging materials/heavy metal salt solution	Amount of stock solution (100 *μ*g/ml) of digest/heavy metal salt solution added to 20 ml of Luria Bertani agar (*μ*l)	Final concentration of heavy metal in Luria Bertani agar (*μ*g/ml)
Vanadium/vanadium pentoxide mercury/mercuric chloride	240	1.2
	280	1.4
	320	1.6
	360	1.8
	400	2.0
	440	2.2

Arsenic/arsenic trioxide	200	1.0
	240	1.2
	280	1.4
	320	1.6
	360	1.8
	400	2.0

Cadmium/cadmium acetate	800	6.4
	1000	6.6
	1200	6.8
	1400	7.0
	1600	7.2
	1800	7.4

**Table 2:** Purity, Codex Alimentarius Commission concentration, and mean minimum inhibitory concentration of heavy metal salts on standard strains of bacteria tested.

Heavy metal salts studied	Heavy metal salt purity	Codex Alimentarius Commission acceptable value (*μ*g/ml)	Minimum inhibitory concentration (*MIC*) ± *standard* *deviation*			
			*E. coli* (*μ*g/ml)	*E. faecalis* (*μ*g/ml)	*K. pneumoniae* (*μ*g/ml)	*P. aeruginosa* (*μ*g/ml)
Vanadium pentoxide	99.96%	1.8	2.0 ± 0.047	2.2 ± 0.0471	2.0 ± 0.047	2.0 ± 0.047
Cadmium acetate	99.87%	7.0	7.0 ± 0.081	7.0 ± 0.081	7.0 ± 0.0471	7.2 ± 0.0471
Mercuric chloride	96.52%	1.4	1.6 ± 0.0471	1.4 ± 0.0471	1.4 ± 0.0471	1.4 ± 0.081
Arsenic trioxide	98.52%	1.6	1.6 ± 0.081	1.4 ± 0.081	1.6 ± 0.0471	1.6 ± 0.081

**Table 3:** Content of heavy metals, CAC concentration, and mean MIC of heavy metal contained in digests of food packaging materials on the standard strains of bacteria tested.

Heavy metals in digests of packaging materials (DPM)	Content of heavy metal in digest (*μ*g/ml)	Codex Alimentarius Commission acceptable value (*μ*g/ml	Minimum inhibitory concentration (*MIC*) ± *standard* *deviation*			
			*E. coli* (*μ*g/ml)	*E. faecalis* (*μ*g/ml)	*K. pneumoniae* (*μ*g/ml)	*P. aeruginosa* (*μ*g/ml)
Vanadium (cardboard)	2.7	1.8	2.2 ± 0.0471	2.2 ± 0.0471	2.0 ± 0.047	2.2 ± 0.0471
Cadmium (sachets)	155	7.0	7.0 ± 0.081	7.0 ± 0.081	7.0 ± 0.081	7.0 ± 0.081
Mercury (sachets)	213	1.4	1.6 ± 0.0471	1.6 ± 0.081	1.6 ± 0.0471	1.4 ± 0.081
Arsenic (medicinal closure)	101.41	1.6	1.6 ± 0.081	1.6 ± 0.0471	1.6 ± 0.081	1.6 ± 0.0471

## Data Availability

All data generated or analysed during this study are included in this article.

## Supplementary Materials

Supplementary Materials Figure S1: sterile Luria Bertani agar used in the study. Figure S2: picture of heavy metal stock solution used in the study. Figure S3: picture of sterile standard heavy metal stock. Figure S4: LB agar plate with heavy metal showing growth of standard strains at different concentrations.
