# Supplementary material for: Evaluation of Minimum Inhibitory Concentration of Heavy Metals Contained in Packaging Material Digest on Prominent Gut Microbiota
Source: Int J Food Sci. 2023 Nov 21;2023:3840795. doi: 10.1155/2023/3840795 (PMC10684320; doi:10.1155/2023/3840795)

**Supplementary material**

**Evaluation of Minimum Inhibitory Concentration of Heavy Metals Contained in Packaging Material Digest on Prominent Gut Microbiota**

**Running title -** Assessing Heavy Metal Minimum Inhibitory Concentration in Packaging Material Digest on Gut Microbiota

^1^Senna Mukhi, ^2^ Biranthabail Dhanashree, ^1^Rukmini Mysore Srikantiah, ^1^Poornima Manjrekar, ^1^Sindhu Harish

^1^ Department of Biochemistry, Kasturba Medical College, Mangalore, Manipal Academy of Higher Education, Manipal, India

^2^ Department of Microbiology, Kasturba Medical College, Mangalore, Manipal Academy of Higher Education, Manipal, India

Correspondence should be addressed to - Dr Dhanashree B; [dhanashree@manipal.edu](mailto:dhanashree@manipal.edu); [dbiranthabail@yahoo.co.in](mailto:dbiranthabail@yahoo.co.in)

**Table of Contents**

Figures S1-S4: Images of the experiment being conducted………….………………. Page no 1-4

Figure S1 -Sterile Luria Bertani agar used in the study

**
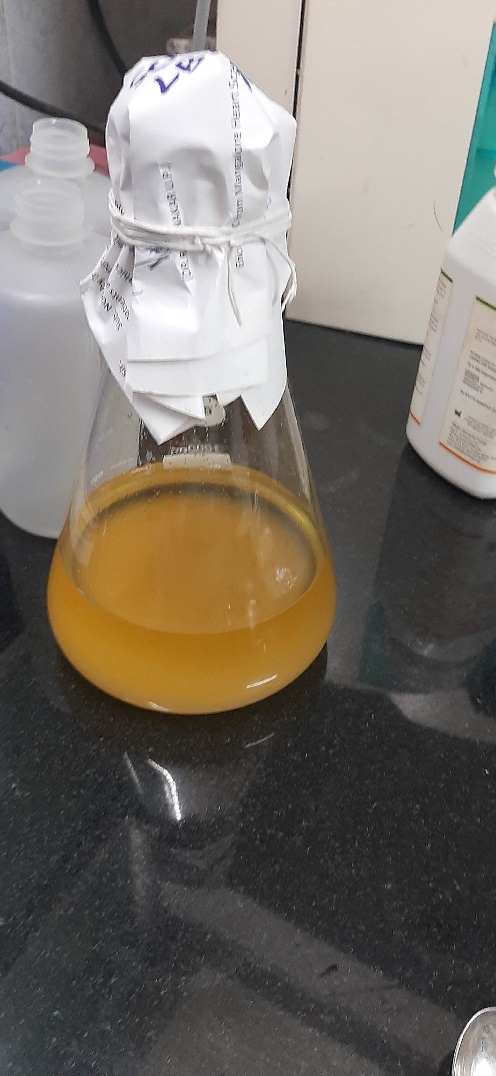
**

**Fi**

Figure S2 – Picture of heavy metal stock solution used in the study

**
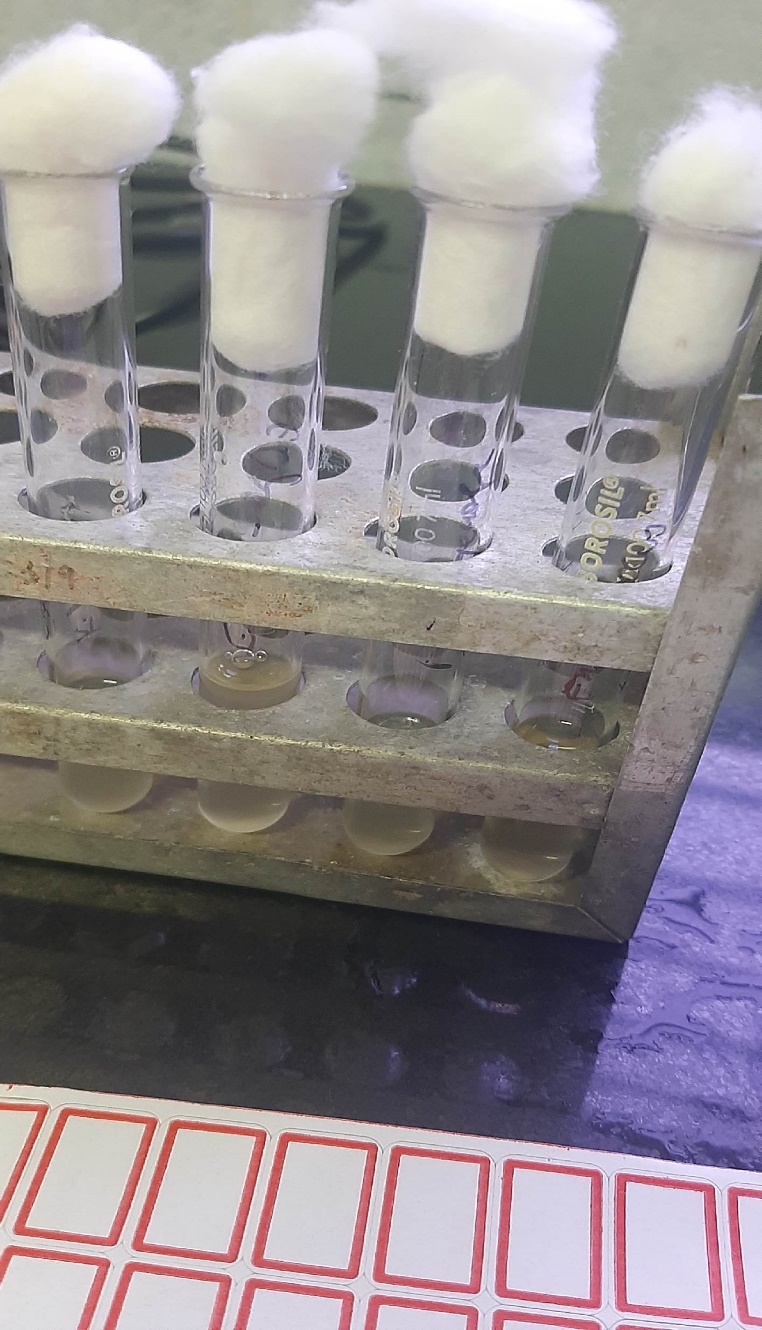
**


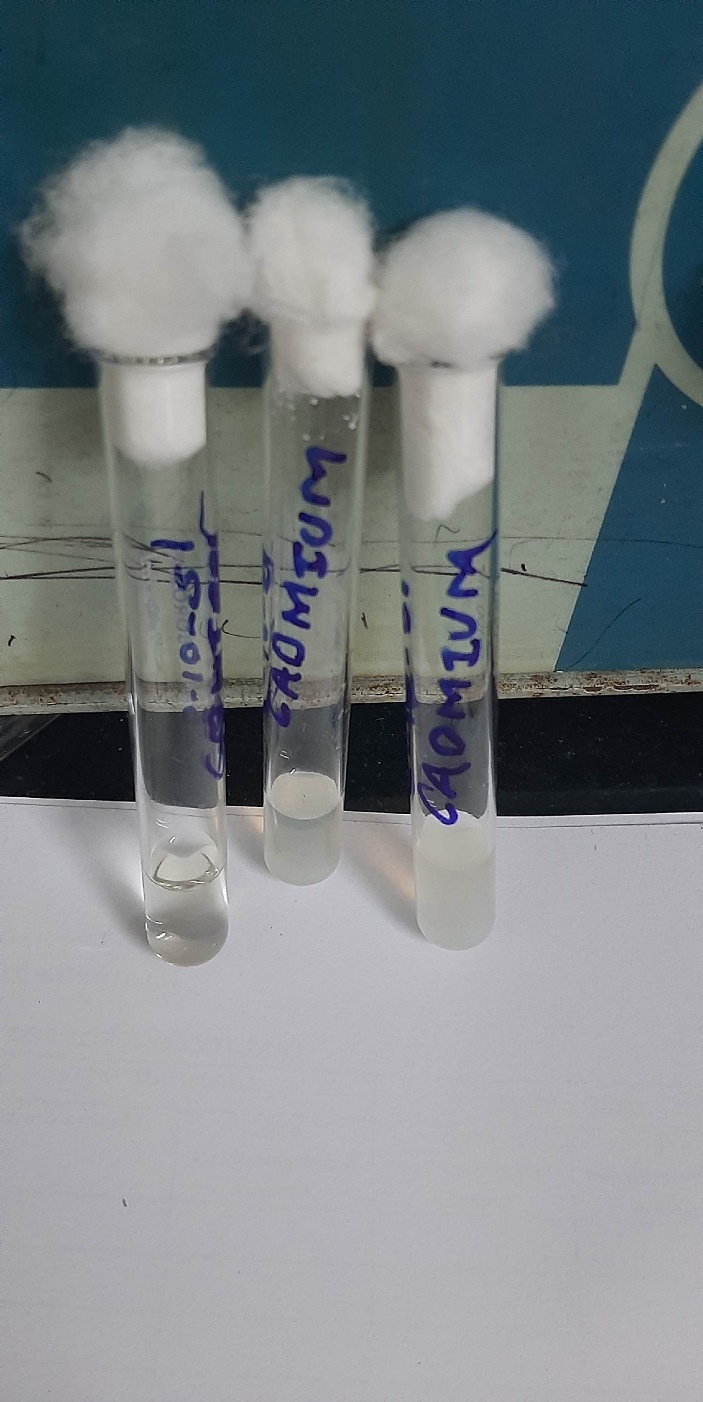
Figure S3- Picture of sterile standard heavy metal stock

F

Figure S4 - LB agar plate with heavy metal showing growth of standard strains at different concentrations


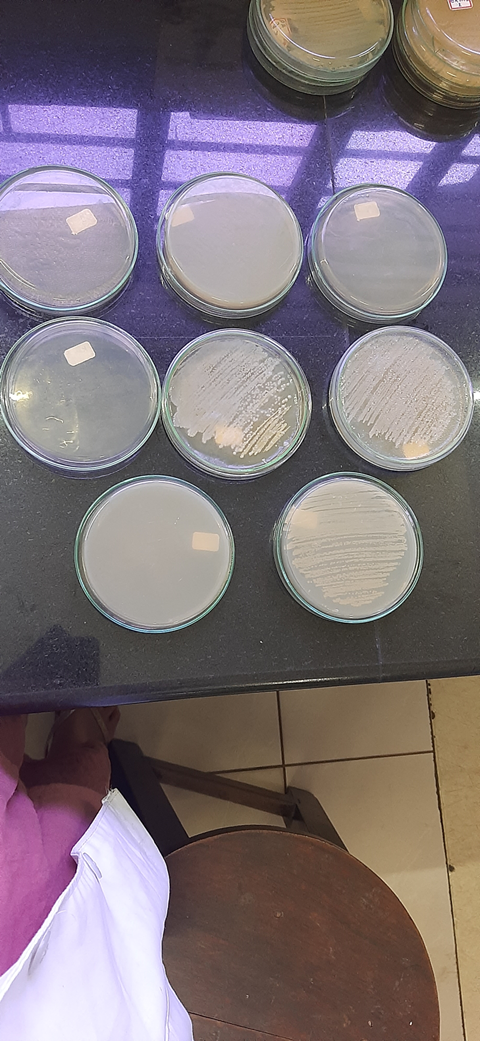

Supplement: Supplementary Materials — Figure S1: sterile Luria Bertani agar used in the study. Figure S2: picture of heavy metal stock solution used in the study. Figure S3: picture of sterile standard heavy metal stock. Figure S4: LB agar plate with heavy metal showing growth of standard strains at different concentrations. [file 3840795.f1.doc]
